# Validation of the Survival Benefits of Metformin in Middle Eastern Patients With Type II Diabetes Mellitus and Colorectal Cancer

**DOI:** 10.1200/JGO.18.00018

**Published:** 2018-06-12

**Authors:** Amal Al Omari, Hadeel Abdelkhaleq, Maysa Al-Hussaini, Rim Turfa, Nour Awad, Manal M. Hassan, Mahmoud A. Alfaqih, Christopher R. Garrett

**Affiliations:** **Amal Al Omari**, **Hadeel Abdelkhaleq**, **Maysa Al-Hussaini**, **Rim Turfa**, and **Nour Awad**, King Hussein Cancer Center, Amman; **Mahmoud A. Alfaqih**, Jordan University of Science and Technology, Irbid, Jordan; and **Manal M. Hassan** and **Christopher R. Garrett**, The University of Texas MD Anderson Cancer Center, Houston, TX.

## Abstract

**Purpose:**

Epidemiologic data from several populations suggest that metformin may decrease cancer risk and mortality in patients with colorectal cancer (CRC) and type II diabetes mellitus (DM). Although type II DM and CRC are major health problems in the Middle East, no investigations have been performed to test the effect metformin has on the outcome of patients with type II DM and CRC who are also treated with metformin.

**Materials and Methods:**

We retrospectively reviewed the medical records of 1,902 patients diagnosed with CRC at King Hussein Cancer Center between January 2004 and December 2012, and identified 349 patients (18%) with type II DM; we censored the data of 28 patients because their antidiabetic medications were unknown. We then categorized these 321 patients into two groups: 192 patients treated with metformin (group A) and 129 patients treated with other antidiabetic medications (group B).

**Results:**

Group A patients had significantly longer overall survival (89 months; 95% CI, 66 to 112 months) and progression-free survival (47 months; 95% CI, 15 to 79 months) than group B patients (overall survival: 36 months; 95% CI, 24 to 48 months; *P* ≤ .001; progression-free survival: 21 months; 95% CI, 13 to 29 months; *P* = .016). After adjustment for age, sex, body mass index, aspirin use, anticholesterol treatment, and CRC stage, group A patients had a 40% reduction in mortality (hazard ratio, 0.58; 95% CI, 0.4% to 0.85%; *P* = .005).

**Conclusion:**

Our results support findings from other populations that patients with diabetes and CRC who are also treated with metformin have better outcomes than those treated with other antidiabetic medications.

## INTRODUCTION

Colorectal cancer (CRC) is the second most common cancer among Jordanian adults, with an annual incidence rate of 13.6% in men and 9.6% in women, and is the second most common cause of cancer-related deaths in Jordan (12.1%).^1^ The prevalence of type II diabetes mellitus (DM) is 20% to 25% in some Middle Eastern countries, which makes it among the highest in the world.^2^ The size of this problem in Jordan, a Middle Eastern country, is not different from other countries in the region. Indeed, in a study of 1,121 Jordanian patients ≥ 25 years of age, the prevalence of type II DM was 17.1%.^3^ Moreover, it is currently estimated that over the past 10 years, the prevalence of diabetes in Jordan significantly increased by 31.5%. The exact cause of this epidemic is currently unknown, but could be related to increased life expectancy and a rapidly changing lifestyle associated with a higher body mass index (BMI) among the Jordanian population; risk factors associated with increased odds of DM and impaired fasting glycemia among Jordanians.^3^

Previous epidemiologic studies suggest that type II DM increases the risk of and mortality rate from cancer, especially GI cancers.^4,5^ A meta-analysis showed that type II DM is associated with a 30% increased risk of CRC in both sexes.^6^ Moreover, patients with CRC and type II DM had a 42% lower 5-year survival rate (all-cause mortality) and were 21% more likely to have CRC recurrence.^7^ The cause of this increased risk is still under investigation, but researchers have hypothesized that the increased levels of insulin and free insulin growth factor 1 may promote proliferation of CRC cells.^8,9^

Metformin, a biguanide that is widely used as a first-line oral antidiabetic medication for type II DM, decreases plasma glucose levels by increasing intracellular glucose uptake. Because insulin resistance and inflammation are the most likely biologic mechanisms underlying DM-related carcinogenesis,^10^ it has been hypothesized that any agent that overcomes insulin resistance would reduce the risk of cancers associated with DM.^11^ Accumulating epidemiologic evidence suggests that metformin is associated with a reduced risk of cancer.^12^ Patients with CRC and DM treated with metformin had a better response rate to cytotoxic chemotherapy and a longer overall survival (OS) than those treated with other antidiabetic medications.^13^ One study demonstrated a higher rate of CRC-related death in patients with type II DM treated with sulfonylureas or insulin compared with those treated with metformin.^14^ Moreover, patients with diabetes and cancer who were treated with neoadjuvant chemotherapy and metformin had a higher pathologic complete response rate (24%) than those treated with other therapies (8%; *P* = .07).^15^

Despite overwhelming data from several populations that support a protective effect of metformin in patients with CRC and DM,^16-18^ there are no studies that investigated this relationship in Jordan. Considering the size of the DM epidemic in Jordan,^3^ the high incidence of CRC,^1^ and the financial burden they place on the economy and health services, we retrospectively assessed the effect of metformin on the survival outcomes of patients with CRC and type II DM in Jordan.

## MATERIALS AND METHODS

### Construction of a Data Base of Patients With CRC at King Hussein Cancer Center

We retrospectively reviewed the medical records of patients with type II DM diagnosed with CRC at King Hussein Cancer Center (KHCC) in Amman, Jordan, from January 1, 2004, to December 31, 2012. Our study was approved by the Institutional Review Board (12 KHCC 49), with a waiver of the written informed consent requirement. All patients were Middle Eastern, including Jordanians and other nationalities coming to KHCC for treatment.

Medical charts of 1,902 patients with CRC were manually reviewed, and patients with CRC and a history of type II DM were identified. All patients had pathologically confirmed colorectal adenocarcinoma (stages I to IV). Other types of carcinoma, such as neuroendocrine carcinoma, were excluded from the final analysis. Structured spreadsheets for data collection and the Microsoft Access data base were developed to assist in reviewing and documenting epidemiologic and clinical factors, including follow-up and survival data.

Of the 1,902 patients with CRC, 349 (18%) had type II DM at the time of their initial consultation at KHCC. Type II DM status before CRC diagnosis was determined from the medical history obtained at the first KHCC visit and by concomitant review of the medical records and medication lists. Patients who developed type II DM after their CRC diagnosis and patients with type I DM were excluded from our analysis. We also censored the data of 28 patients because their antidiabetic medication history was unknown.

Patient demographics and clinical characteristics, including age at the time of diagnosis, sex, BMI, date of CRC diagnosis, follow-up duration, and adjuvant, neoadjuvant, and metastatic cancer therapies were all obtained from the medical charts. BMI categories were defined as follows: underweight/normal (BMI < 24.9 kg/m^2^), overweight (BMI range, 25 to 29.9 kg/m^2^), and obese (BMI ≥ 30 kg/m^2^). Follow-up data for survival analysis were available for 277 patients. The type of antidiabetic medications used before CRC diagnosis, glycosylated hemoglobin levels, aspirin use, and anticholesterol medications were also recorded.

Data on CRC diagnosis, including tumor site, size, pathologic stage (TNM), grade of differentiation, and lymphovascular invasion were collected from the pathology reports. The pathologic response rates after neoadjuvant chemoradiation therapy for rectal cancers and perioperative cytotoxic chemotherapy for hepatic metastases were evaluated semiquantitatively as follows: complete response, no residual tumor in the primary site or in the lymph nodes; partial response, < 50% of the primary tumor was viable; and no response, ≥ 50% of the tumor was viable or the tumor had metastasized to the lymph nodes. The Response Evaluation Criteria in Solid Tumors (RECIST) was used to evaluate the response to palliative cytotoxic chemotherapy for CRC with distant metastasis. The response to the last line of palliative chemotherapy was confirmed by a computed tomography scan performed immediately after the last cycle for which the RECIST response was recorded.

### Statistical Analysis

All clinical and epidemiologic data were merged, and descriptive statistics were generated. Continuous data were compared using the Student *t* test, and categorical data were compared using the χ^2^ test. OS was defined as the time between CRC diagnosis and death or end of follow-up (censored data). Progression-free survival (PFS) was defined as the time between CRC diagnosis and recurrence or disease progression, or end of follow-up (censored data). We categorized patients into two groups: patients treated with metformin (group A) and patients treated with other antidiabetic medications (group B). The Kaplan-Meier method was used to estimate median survival duration, and the log-rank test was used to determine the significance of differences in survival duration between the two patient groups. A multivariable Cox proportional hazards regression model was used to calculate hazard ratios (HRs) and 95% CIs with a backward stepwise selection procedure, considering the clinical covariables of CRC survival. *P* values ≤ .05 were considered statistically significant. All statistical analyses were performed using IBM SPSS software, version 21.0 (SPSS, Chicago, IL).

## RESULTS

The final study cohort consisted of 321 patients; the mean age ± standard deviation was 61.7 ± 9 years, and the median age was 62 years (range, 36 to 82 years). The male-to-female ratio was approximately 1.5:1. Of the 321 patients with known antidiabetic medication history, 192 (60%) were treated with metformin (group A): 58 (18%) were treated with metformin alone, 15 (5%) were treated with metformin and insulin, and 119 (37%) were treated with metformin and another oral antihyperglycemic agent. Group B included 129 patients (40%) who were not treated with metformin. Overall, metformin use increased over time, from 26% in 2004 to 85% in 2012.

Table 1 lists the demographic and clinical features of these 321 patients, including sex, age at diagnosis, cancer stage, and cancer site, which did not differ significantly between group A and group B. However, group A patients were significantly more likely to be overweight or obese (77%) than group B patients (59%; *P* = .005). Anticholesterol medication use at the time of first evaluation at KHCC was documented in 111 patients (35%) and was higher in group A (42%) than in group B (24%; *P* = .001). Of the 321 patients, 141 (44%) used aspirin at the time of the initial consultation at KHCC. Aspirin use was higher in group A (50%) than in group B (36%; *P* = .014). In addition, group A patients showed significantly better glycosylated hemoglobin levels than group B patients (53% *v* 38%; *P* = .028).

**Table 1:**
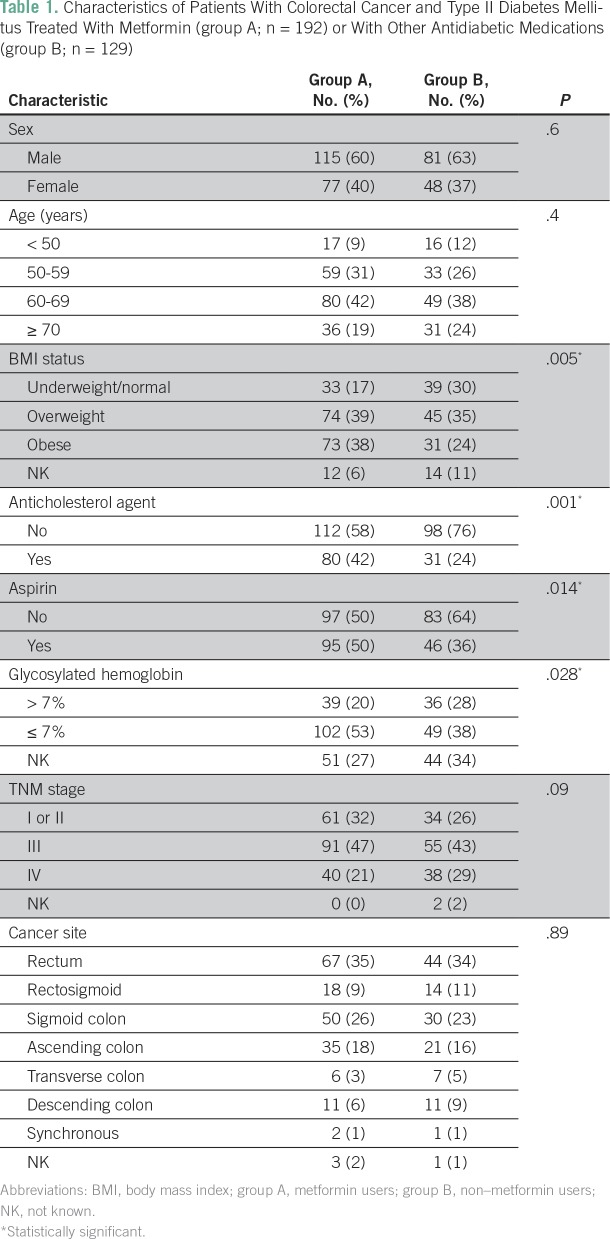
Characteristics of Patients With Colorectal Cancer and Type II Diabetes Mellitus Treated With Metformin (group A; n = 192) or With Other Antidiabetic Medications (group B; n = 129)

**Fig 1:**
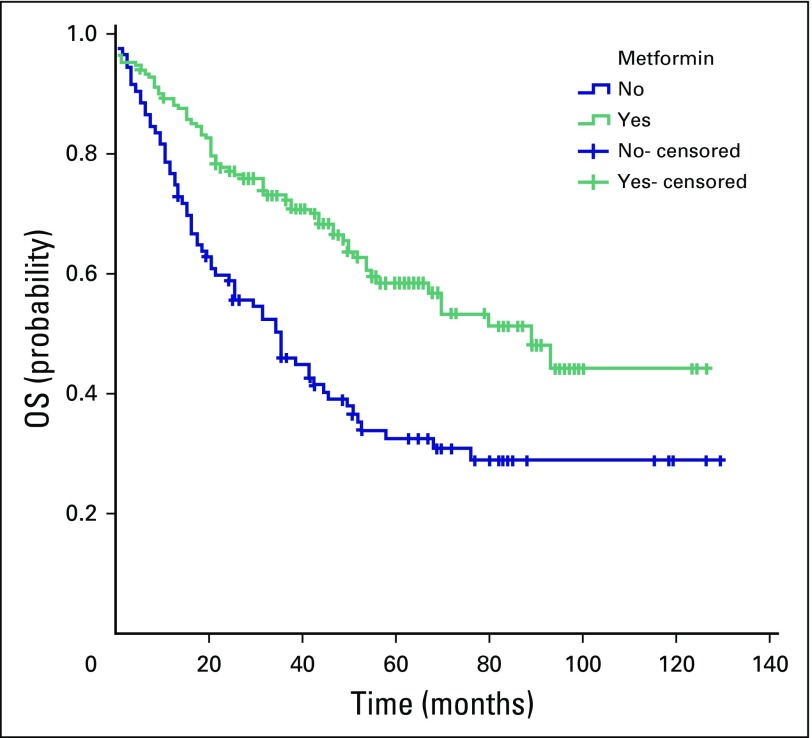
Comparison of median overall survival (OS) between patients with colorectal cancer and type II diabetes mellitus treated with metformin (group A, green line) and those treated with other antidiabetic medications (group B, blue line). The median OS was 89 months (95% CI, 66 to 112 months) for group A and 36 months for group B (95% CI, 24 to 48 months; *P* < .001). The 5-year OS rate was 58% for group A and 32% for group B.

The median follow-up duration was 37 months (range, 0 to 129 months). Death was confirmed in 142 patients. For all patients, the 5-year survival rate was 49**%**, and the median OS was 53 months (95% CI, 39.6 to 66.4 months). The median OS was 89 months (95% CI, 66 to 112 months) for group A and 36 months (95% CI, 24 to 48 months) for group B (*P* < .001). The 5-year survival rate was 58% for group A and 32% for group B (Fig 1).

**Fig 2:**
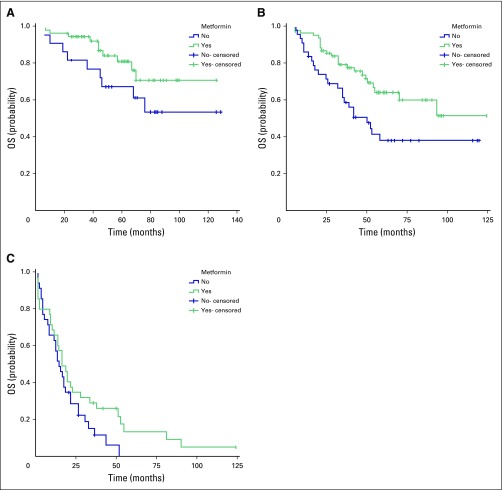
Comparison of median overall survival (OS) between patients with colorectal cancer (CRC) and type II diabetes mellitus treated with metformin (group A, green line) and those treated with other antidiabetic medications (group B, blue line) stratified by TNM stage. (A) The median OS for patients with stage I or II CRC in group A and group B was not reached; *P* = .133. The 5-year OS rate was 81.0% for group A and 67.4% for group B. (B) For patients with stage III CRC in group A, the median OS time was not reached, and in group B, the median OS was 50 months (95% CI, 32 to 68 months; *P* = .014). The 5-year OS rate was 64% for group A and 38% for group B. (C) For patients with stage IV CRC, the median OS was 16 months (95% CI, 11 to 21 months) in group A and 14 months (95% CI, 9 to 19 months) in group B (*P* = .105). The 5-year OS rate for group A was 12.7% and 0% for group B.

However, when stratified by TNM staging, the difference in median OS between the two groups was statistically significant only in patients with stage III CRC (*P =* .014). Although the median OS for patients with stage III CRC in group A was not reached, the median OS for those in group B was 50 months (95% CI, 32 to 68 months; Fig 2B). Group A patients with stage IV CRC had slightly longer OS (16 months; 95% CI, 11 to 21 months) than those in group B (14 months; 95% CI, 9 to 19 months; Fig 2C), but the difference was not significant (*P* = .11). The median OS for patients with stage I to II CRC was not reached for either group; however, the OS rate was higher in group A than in group B at all time points (Fig 2A), with no statistical significance (*P* = .13).

Adjusting for age, sex, BMI, aspirin use, anticholesterol treatment, and initial stage of CRC, metformin use was found to be associated with a significant 40% reduced risk of mortality (HR, 0.58; 95% CI, 0.4 to 0.85; *P =* .005; Table 2). Overweight patients had significantly longer median OS than underweight/normal patients (70 months [95% CI, 46 to 94 months] versus 33 months [95% CI, 11 to 55 months]; *P* = .004). The median OS was not reached for obese patients. On multivariable analysis with BMI as a continuous variable, patients with high BMI had a lower risk of death than those with low BMI (HR, 0.94; 95% CI, 0.9 to 0.98; *P* = .006; Table 2).

**Table 2:**
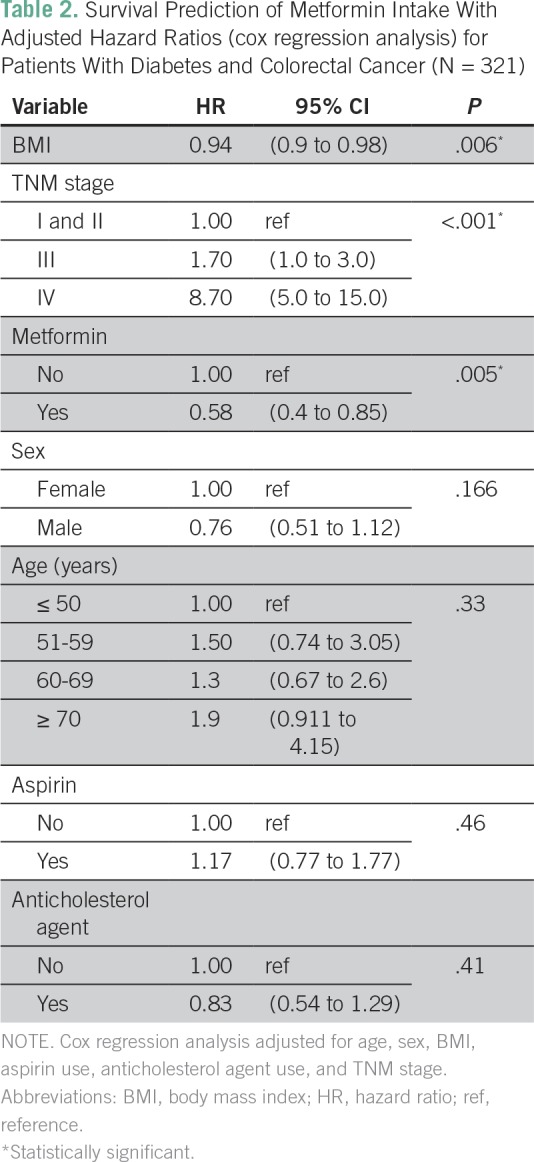
Survival Prediction of Metformin Intake With Adjusted Hazard Ratios (cox regression analysis) for Patients With Diabetes and Colorectal Cancer (N = 321)

**Fig 3:**
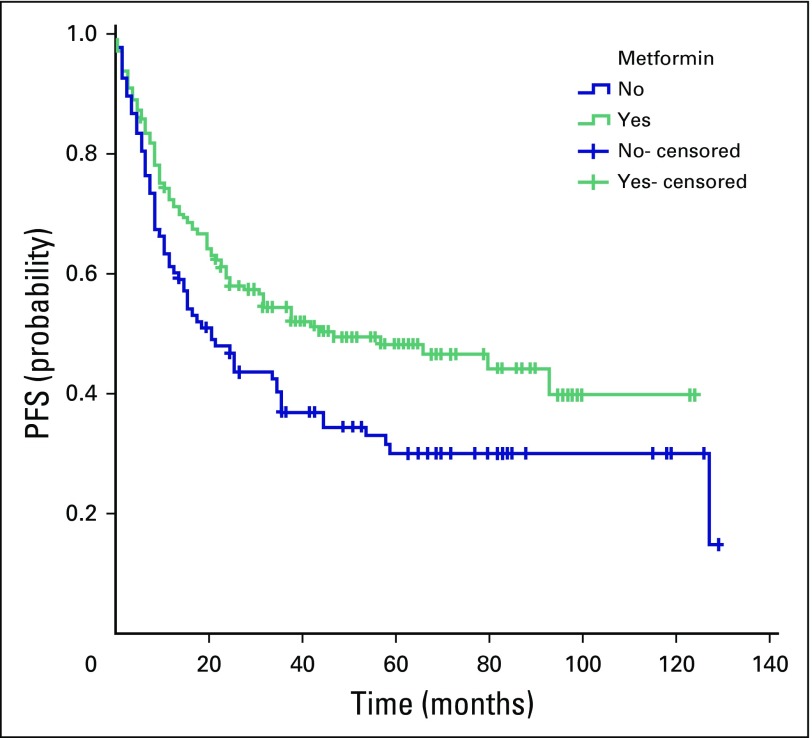
Comparison of the median progression-free survival (PFS) between patients with colorectal cancer and type II diabetes mellitus treated with metformin (group A, green line) and those treated with other antidiabetic medications (group B, blue line). In group A, the median PFS was 47 months (95% CI, 15 to 79 months), and in group B, the median PFS was 21 months (95% CI, 13 to 29 months; *P* = .016). The 5-year PFS rate for group A was 48% and 30% for group B.

Disease progression, recurrence, or death was observed in 179 patients. The median PFS for all patients was 34 months (95% CI, 23 to 45 months). The median PFS in group A (47 months; 95% CI, 15 to 79 months) was significantly longer than in group B (21 months; 95% CI, 13 to 29 months; *P =* .016; Fig 3). When patients were categorized according to tumor stage, the beneficial effect of metformin use was not observed in patients with stage I to II or stage IV tumors. Patients with stage III CRC in group A had longer median PFS (66 months; 95% CI, 25 to 107 months) than those in group B (35 months; 95% CI, 9 to 61 months; *P* = .067).

Patients with distant metastasis who received palliative cytotoxic chemotherapy had a significantly better radiologic (RECIST) response rate (ie, complete, partial, or stable disease) in group A (26 of 54; 48%) than in group B (11 of 44, 25%; *P* = .013; Table 3). The pathologic response rate in patients with rectal cancer who received neoadjuvant chemoradiotherapy did not differ significantly between group A and group B.

**Table 3:**
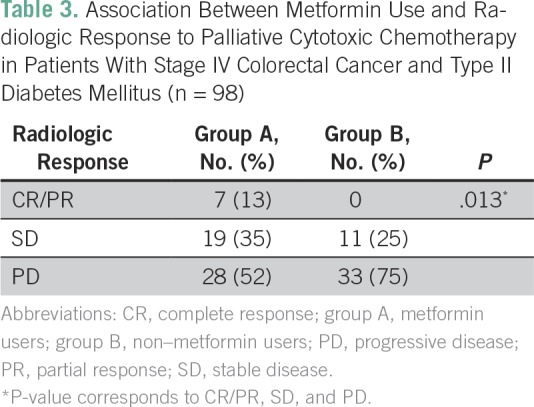
Association Between Metformin Use and Radiologic Response to Palliative Cytotoxic Chemotherapy in Patients With Stage IV Colorectal Cancer and Type II Diabetes Mellitus (n = 98)

## DISCUSSION

To the best of our knowledge, our study is the first to demonstrate the beneficial effect of metformin on survival in Middle Eastern patients with type II DM and CRC. In this population, the prevalence of type II DM was 18%, similar to what was reported previously in Jordanians,^3^ but much higher than other populations.^18^ The use of metformin was associated with longer median OS and PFS. Although the beneficial effect of metformin was seen in patients regardless of CRC stage, metformin’s effect on median OS and PFS was statistically significant (OS) or close to significance (PFS) only in patients with stage III CRC. Metformin use was associated with a 40% improvement of OS after adjusting for other clinically relevant variables.

Cancer and type II DM share many risk factors, such as limited physical activity, high BMI, a diet high in saturated fat, and smoking. Diabetes and hyperglycemia are associated with an increased risk of developing cancers of the pancreas, liver, colon, breast, and uterus.^19^

A growing number of human observational studies have suggested that metformin use is associated with improved OS and cancer-specific survival in patients with type II DM and cancers of the breast,^20,21^ ovary,^22^ liver,^23^ pancreas,^24^ and lung,^25,26^ and CRC.^27^ A meta-analysis of 20 publications that involved 13,008 patients with cancer with concurrent type II DM suggested that metformin could be the drug of choice in these patients.^28^ Metformin, one of the most widely used drugs for treating type II DM, has been reported to have a potential anticancer effect. In a meta-analysis of 71 studies that assessed the effect of metformin on cancer incidence and mortality, Gandini et al^29^ concluded that metformin is associated with reduced cancer incidence and mortality. Consistent with our findings, a recent meta-analysis of six cohort studies involving 2,461 patients with CRC with type II DM demonstrated that treatment with metformin reduced the risk of death from all causes by 44% and the risk of CRC-specific death by 34% compared with other antidiabetic medications.^17^ In our study, metformin treatment was also associated with prolonged PFS. The 5-year PFS rate was 48% for group A and 30% for group B.

Most of our patients with stage III CRC were treated with adjuvant cytotoxic chemotherapy. Our findings may suggest combining metformin with adjuvant chemotherapy to treat locally advanced CRC. A randomized clinical trial is needed to determine the efficacy of this combination therapy. Several phase II clinical trials (https://clinicaltrials.gov) include metformin as a treatment of CRC (mostly in combination therapies) because the combination of metformin with various chemotherapeutic agents has shown a synergistic effect in various tumor types.^30^

In a previous study, metformin was associated with increased cancer-specific survival only in the adjuvant chemotherapy setting, regardless of cancer stage.^13^ In an experimental study, metformin was found to selectively target cancer stem cells and to act synergistically with chemotherapy.^31^ Metformin may also reduce the effective doses of chemotherapeutic agents needed to treat various cancers.^32^

Our patients with advanced CRC in group A had a better radiologic response to palliative cytotoxic chemotherapy than those in group B. However, investigators from another study reported no significant difference in tumor response, change in target lesion size, PFS rate, or OS rate among 81 patients with diabetes with stage IV CRC treated with metformin and palliative chemotherapy.^33^

Metformin may have a direct effect on tumor cells independent of its effects on circulating insulin and insulin growth factor 1. At the molecular level, the principal target of metformin is AMP-activated protein kinase (AMPK). This kinase is an energy sensor and is critical for the effect of metformin on hepatic gluconeogenesis by disrupting mitochondrial ATP production.^34^ This leads to an increased cellular AMP:ATP ratio.^35^ Activated AMPK inhibits mammalian target of rapamycin, a protein that is activated by insulin growth factor 1 and that drives metabolism, growth, and proliferation of cancer cells.^36,37^ In vitro and preclinical studies showed that metformin inhibits the mammalian target of rapamycin pathway either dependently or independently of AMPK activation, leading to decreased cellular proliferation^38^ and increased sensitivity to therapy.^39^ In CRC, activated AMPK has been associated with a good prognosis, providing a link among metformin, AMPK, and improved outcome in patients with this disease.^40^

This retrospective analysis is limited by the fact that the effect of metformin dose intensity and duration of metformin treatment, the date of onset of type II DM, and the potential added effect of other antidiabetic medications could not be evaluated. Although group A patients seemed to be more likely to seek care because of the significant difference in aspirin use, anticholesterol use, and the percentage with more controlled glycosylated hemoglobin levels compared with group B (Table 1), there was no selection bias in our study design because all records of patients with CRC during the study period of January 1, 2004, to December 31, 2012, were reviewed, and those with a history of type II DM were identified and included in the analysis. Also, glycosylated hemoglobin levels were known in only a small fraction of patients in both groups. Knowing the retrospective nature of the study design, these limitations should be taken into consideration when interpreting the results. We controlled for the effect of aspirin and anticholesterol agent use in the multivariable Cox regression analysis, and the effect of metformin on survival was significant despite these covariables. Nonetheless, prospective studies are needed to validate the potential survival benefit associated with the use of metformin in patients with type II DM and CRC to assess the effect of metformin dose intensity and duration on survival outcomes and to address the safety and efficacy of metformin in patients with CRC without diabetes.

Our results support prior findings from the literature in a new unstudied population of Middle Eastern patients. Our patients with CRC and type II DM treated with metformin had a lower risk of overall mortality and longer PFS than those treated with other antidiabetic medications.
